# Analysis of the absorbed constituents and mechanism of liquidambaris fructus extract on hepatocellular carcinoma

**DOI:** 10.3389/fphar.2022.999935

**Published:** 2022-08-30

**Authors:** Shuai Wang, Xin-Xin Yang, Tian-Jiao Li, Lin Zhao, Yong-Rui Bao, Xian-Sheng Meng

**Affiliations:** ^1^ College of Pharmacy, Liaoning University of Traditional Chinese Medicine, Dalian, China; ^2^ Liaoning Multi-Dimensional Analysis of Traditional Chinese Medicine Technical Innovation Center, Dalian, China; ^3^ Liaoning Province Modern Chinese Medicine Research Engineering Laboratory, Dalian, China

**Keywords:** liquidambaris fructus, hepatocellular carcinoma, absorbed constituents, pharmacology and efficacy, mechanism of action

## Abstract

**Background:** Hepatocellular carcinoma (HCC) refers to one of the top 10 cancers in terms of morbidity and mortality globally, seriously influencing people’s lives. First recorded in Compendium of Materia Medica, liquidambaris fructus (LF) generates definite anti-liver tumor effect. However, its effective substances and mechanism remain to be elucidated.

**Methods:** Serum pharmacochemistry and UPLC-QTOF-MS technologies were employed to explore the plasma of rats after intragastric administration of liquidambaris fructus extract (LFE) in order to find the active ingredients. Subsequently, DEN-induced rat liver cancer model was established with the purpose of investigating the anti-tumor activity of LFE from physiological, pathological and biochemical aspects. Finally, non-target metabonomics combined with q-PCR and Western blot methods were adopted for revealing the mechanism.

**Results:** Totally 11 prototype blood transfused ingredients, including imperatorin and phellopterin were detected. LFE presents excellent impact on enhancing the quality of life, prolonging the life cycle, reducing inflammatory reaction, protecting hepatocytes, improving body immunity and killing liver tumor cells. Altogether 82 endogenous differential metabolites were found in metabonomics, suggesting that LFE can treat HCC by acting on key targets of PTEN/PI3K/Akt pathway and fatty acid metabolism. Further research also verified that LFE can upregulate the relative expression levels of PTEN, PDCD4, Caspase 9, Caspase 3, Bax and Bad as well as lower the relative expression levels of PI3K, AKT, VEGFA and Bcl-2.

**Conclusion:** This study revealed the pharmacodynamic material basis of LFE in the treatment of HCC, and from the perspective of metabolomics proved that the effects of inhibiting the growth of tumor cells, promoting tumor cell apoptosis, reducing inflammatory reaction, protecting hepatocytes, improving the survival state of tumor rats, and prolonging the life cycle are related to its impact on PTEN/PI3K/Akt, fatty acid metabolism and other key signal pathways.

## Introduction

Cancer is one of the diseases causing great harm to human health. In accordance with the latest report “Global Cancer Statistics 2020” released by the World Health Organization (WHO), the number of new cancer patients in the world in 2020 reached 19.29 million and that of deaths was 9.96 million (Sung et al., 2021). Among them, hepatocellular carcinoma (HCC) is known as the “king of cancer,” which is among the top ten cancers globally in terms of incidence rate and mortality (Bao et al., 2017). Although HCC ranks only the sixth in the global cancer incidence rate ranking (910,000 people, 4.7%), it is characterized by a high mortality rate (830,000 people, 8.3%) due to the fact that it is mostly found in the middle and late stage and has no obvious pain (Nadarevic et al., 2022). At present, in clinical treatment, most patients with liver cancer are discovered to be in the middle and advanced stage, and exhibit abnormal liver function with systemic metastasis, and thus they cannot accept radical surgery. Nevertheless, there are some major problems in the process of radiotherapy and chemotherapy, including poor specificity, strong drug resistance, and large toxic and side effects (Guo et al., 2019). It has always been the primary problem of anti-tumor drug research and development to find a highly specific drug for HCC that can not only prolong the life cycle and guarantee the quality of life, but also assist in the treatment.

In China, Traditional Chinese medicine (TCM) has been adopted for thousands of years, attracting more and more researchers’ attention because of its unique curative effect and great development potential. In addition, the multi-component and multi-target characteristics of TCM can potentially enhance the curative effect while reducing the toxicity and side effects, especially suitable for the treatment of complex diseases (Huang et al., 2020). Liquidambaris fructus (LF), a Traditional Chinese medicine, was first recorded in Compendium of Materia Medica. It is the dried and mature fruit sequence of *Liquidambar formosana* Hance, a plant in the family hamhamica (Qian et al., 2020). It has porous fruit, which goes to the liver and kidney channels, and has the effect of passing twelve channels. Modern pharmacological studies demonstrate that LF possesses the functions of liver protection, anti-tumor and anti-inflammation (Yang X. X. et al., 2020; Li et al., 2021), and has inhibitory impacts on various tumor cells (Fang and Ji, 2019; Qian et al., 2020), especially liver cancer (Yang Y. P. et al., 2020). In addition, our previous studies have reported that liquidambaris fructus extract (LFE) can hinder cancer cells proliferation, and trigger the cell cycle arrest and apoptosis to exert the anti-tumor role (Yang X. X. et al., 2020; Zhang et al., 2020). And the main chemical components of LFE were identified by chemical separation. However, the active ingredients and precise mechanism underlying remains poorly understood.

Therefore, based on the clear study of the chemical components contained in LFE in the early stage, the current experiment adopts the research method of serum pharmaceutical chemistry and UPLC-QTOF-MS technology for analyzing its absorbed components into the blood, aiming to clarify its active components that play the anti-tumor role (Wang et al., 2021; Xu et al., 2021). Furthermore, the anti-liver cancer effect of LFE was comprehensively evaluated from the changes of physiological, pathological and biochemical indexes. On this basis, non-target metabonomics, q-PCR and Western blot techniques were employed to investigate the key targets and pathways of LFE. Specifically, this study clarified the effective substances, deeply revealed the mechanism of LFE against HCC based on the definite anti-liver tumor effect *in vivo*, aiming to provide a scientific explanation for the clinical application of LFE.

## Materials and methods

### Chemicals and reagents

Dried mature inflorescences of *Liquidambar formosana* Hance was obtained from Anguo Qimei medicinal materials Co., Ltd. (Anguo, China). MS grade methanol and acetonitrile were bought from Merck (Darmstadt, Germany). Diethylnitrosamine (DEN) was provided by Sigma (United States). Rat alpha-fetoprotein (AFP) ELISA kit, Rat alanine transaminase (ALT) ELISA kit, Rat aspartate amino-transferase (AST) ELISA kit, Rat tumor necrosis factor-α (TNF-α) ELISA kit were acquired from Shanghai Langdon Biotechnology Co., Ltd. (Shanghai,China).

### Animals

Totally ninety healthy male Sprague-Dawley (SD) rats, weighing (200 ± 20) g, were purchased from Liaoning Changsheng Biotechnology Co., Ltd. with the Certificate No. SCXK (Liao) 2015-0001. After 1 week of adaptation in the free feeding and drinking environment, the experiment was performed. In addition, all the experiments were carried out following the approved animal protocols and guidelines proposed by Medicine Ethics Review Committee for animal experiments of Liaoning University of Traditional Chinese Medicine with approval number: 2020YS013(KT)-013-01.

### Preparation of liquidambaris fructus extract

LF was air-dried, and extracted with 10 times ethyl acetate under reflux for 1 h. Then, the solvent was recovered, and dried LFE was obtained. The extraction ratio is 3.05%.

### Preparation of plasma samples for pharmacochemical analysis

At random, sixteen SD rats were categorized into blank group and administration group, eight rats per group. Besides, the administration group was given 0.27 g/kg/day LFE by gavage (10 times of the clinical dosage, according to crude drug), and the blank group was given the same amount of tertiary water, twice a day for 3 consecutive days. Besides, 12 h before the last administration, fasting and drinking were forbidden. Then, 1 h after the last administration, 1% pentobarbital sodium solution was employed for anesthesia, blood was gathered from hepatic portal vein. After standing at 4°C for 30 min, it was centrifuged at 3,500 rpm for 10 min and plasma was collected. Taking plasma 200 μl, add 5 times methanol:acetonitrile (1:1, V/V) solvent, vortex shock for 2 min, ultrasonic extract for 1 min, freeze stand at −20°C for 10 min, centrifuge at 12,000 rpm for 15 min at 4°C to precipitate protein. Then, take the supernatant, vacuum centrifuge at 1,200 rpm, add 50 μl methanol:acetonitrile (1:1, V/V) solvent redissolved, eddy shock for 2 min, ultrasonic extract for 1 min and centrifuge at 12,000 rpm for 10 min at 4°C. Finally, the supernatant was absorbed for analysis.

### Chemical components absorbed into rat plasma

In this study, UPLC-QTOF-MS technology was employed to detect the chemical components absorbed into rat plasma. Besides, the plasma samples were explored on an Agilent-1290 UPLC system coupled with the Agilent-6550 QTOF mass spectrometry (Agilent Technologies, Inc., United States). Chromatographic analysis was performed on an Agilent Poroshell 120 column (100 mm × 4.6 mm, 2.7 μm) with flow rate 0.8 ml/min, column temperature 30°C, injection volume 3 μl. In the positive ion mode, the system of 0.1% formic acid aqueous solution (A)-acetonitrile:methanol (95:5) (B) was used, and the gradient elution conditions were 0–5 min, 5%–20% B; 5–60 min, 20%–100% B. While in the negative ion mode, the mobile phase was water (A)-acetonitrile:methanol (95:5) (B) with gradient elution procedure: 0–17 min, 5%–40% B; 17–22 min, 40%–65% B; 22–45 min, 65%–95% B; 45–50 min, 95%–100% B; 50–60 min, 100%–100% B. The MS experiment was performed on a dual spray ion source (Dual AJS ESI) in positive and negative ion modes, with Vcap 4,000 V, Drying Gas Flow 13 L/min, Drying Gas Temp 250°C, Neulizer pressure 45 psig, Sheath Gas Temp 350°C, Sheath Gas Flow 11 L/min, Fragmentor 125 V, and the MS data were collected from m/z 100 to 1,000.

### Construction of DEN-induced hepatocellular carcinoma rat model and liquidambaris fructus extract treatment

Rat HCC model was established by DEN induction method. Seventy-four healthy SD rats were randomly classified into blank group (*n* = 10) and model group (*n* = 64). The model group was given 1% DEN solution by gavage at a dose of 70 mg/kg and the blank group was given the same proportion of normal saline once weekly for a total of 14 weeks. After 6 weeks of modeling, the rats in the model group were randomly classified into five groups, including model group (*n* = 20), cyclophosphamide group (*n* = 24) (10 mg/kg/day), LFE low dose group (*n* = 10) (0.27 g/kg/day) and LFE high dose group (*n* = 10) (0.81 g/kg/day). Different doses of liquid were given to each administration group by gavage. The blank group and model group were given equal volumes of normal saline once daily until the 16th weekends.

After the last administration in the 16th week, the mortality rate of each group was calculated. Besides, the remaining rats were sacrificed with 3% pentobarbital. Blood was collected in two parts. Serum was used to detect the contents of AFP, ALT, AST and TNF-α, and plasma was taken for performing metabonomic analysis. Besides, liver, spleen and thymus were excised and weighed to calculate the liver index, spleen index as well as thymus index. The calculation formula is expressed as follows: liver body ratio = (liver weight/body weight) × 100, thymus index = (thymus weight/body weight) × 100, spleen index = (spleen weight/body weight) × 100. Part of liver tissue was taken out and immersed in fixative solution. Histopathological sections were prepared by HE staining. In addition, electron microscope was used to observe changes in tissue structure, tumor cell density, apoptosis and necrosis degree.

### Metabonomics analysis

Non-target metabonomics analysis was performed on the plasma of rats with therapeutic effect of LFE on DEN-induced liver cancer. 200 μl plasma was mixed with 800 μl precooled methanol and placed in a vortex for 2 min, which was then centrifuged at 10,000 rpm for 10 min at 4°C. Subsequently, the supernatant was dried with nitrogen and 100 μl precooled methanol was added before being placed in a vortex for 2 min and centrifuged at 10,000 rpm for 2 min at 4°C. In addition, the supernatant was taken for carrying out UPLC-QTOF-MS analysis.

Agilent Poroshell 120 EC-C18 (2.1 mm × 100 mm, 1.9 μm) column was employed to perform chromatographic analysis. Column temperature is 30°C and the flow rate is 0.4 ml/min. The mobile phase is A: 0.1% formic acid-water, B: acetonitrile:methanol = 9:1. Injection volume 2 μl, while gradient elution: 0–2 min, 3%–3% B; 2–5 min, 3%–10% B; 5–6 min, 10%–55% B; 6–10 min, 55%–80% B; 10–22 min, 80%–95% B; 22–30 min, 95%–100% B; 30–35 min, 100%–100% B. Moreover, positive and negative ion modes were also adopted for mass spectrometry analysis. Other conditions were consistent with blood component analysis, except that the neulizer pressure was 30 psig, m/z range was 100–1,500, and the Vcap of negative ion mode was 3,500 V.

### Multivariate data analysis

The plasma metabolism profiles of rats in different groups were normalized through the use of Profinder B.08.00 software including peak detection and peak alignment, and then converted into. Cef files. Subsequently, they were imported into Mass Profiler Professional (MPP) B.14.00 for principal component analysis (PCA), partial least squares discriminant analysis (PLS-DA), T-test analysis and variance analysis. With *p* < 0.05 and Fold change value greater than 2 as the criterion, endogenous differential metabolites were screened. The accurate quality and chemical composition of differential metabolites were obtained using ID Browser function. Then, the above data and fragment spectral data were compared with the standard data in the database to identify the endogenous differential metabolites. Through the biological function analysis of metabolites, the metabolic pathway of LFE to interfere with liver cancer was discovered. In the meanwhile, the mechanism of action of LFE against liver cancer was discussed.

### q-PCR analysis

Human HCC SMMC-7721 cells (Wuhan Bode Bioengineering Co., Ltd., Wuhan, China) were routinely cultured in 6-well plates. When the cell density was approximately 70%, LFE containing culture medium with the concentration of 0.5 mg/ml was used for drug intervention. After 36 h, total RNA was extracted from cells with TRIzol (TransGen Biotech, Beijing, China). TransScript First-Strand cDNA Synthesis SuperMix kit (TransGen Biotech, Beijing, China) was employed to synthesize the first strand of cDNA. TransStart Top Green qPCR SuperMix kit (TransGen Biotech, Beijing, China) was performed to amplify the target gene. The whole reflection was conducted on a Real-time Thermal Cycler 5,100 (Thermo Fisher Scientific Inc., Waltham, MA, United States). In addition, the primer sequence of the target genes were presented as follows: PTEN forward primer 5′-TGT​AAA​GCT​GGA​AAG​GGA​CGA-3′ and reverse primer 5′-GGG​AAT​AGT​TAC​TCC​CTT​TTT​GTC-3′, PI3K forward primer 5′-CCA​GGG​AAA​TTC​TGG​GCT​CC-3′ and reverse primer 5′-TGT​ATT​CAG​TTC​AAT​TGC​AGA​AGG​A-3′, AKT forward primer 5′-CAG​GAT​GTG​GAC​CAA​CGT​GA-3′ and reverse primer 5′-AAG​GTG​CGT​TCG​ATG​ACA​GT-3′, VEGFA forward primer 5′-CTG​TCT​AAT​GCC​CTG​GAG​CC-3′ and reverse primer 5′-TTA​ACT​CAA​GCT​GCC​TCG​CC-3′, PDCD4 forward primer 5′-ACC​CTG​CAG​ATC​CTG​ATA​ACT-3′ and reverse primer 5′-TCC​TTA​GTC​GCC​TTT​TTG​CCT-3′, Bax forward primer 5′-TTG​CTT​CAG​GGT​TTC​ATC​C-3′ and reverse primer 5′-GAC​ACT​CGC​TCA​GCT​TCT​TG-3′, Bcl-2 forward primer 5′-AGT​ACC​TGA​ACC​GGC​ACC​T-3′ and reverse primer 5′-CAG​CCA​GGA​GAA​ATC​AAA​CA-3′, Bad forward primer 5′-CAG​ACC​CGG​CAG​ACA​GAT​GAG-3′ and reverse primer 5′-CTC​TGG​GCT​GTG​AGG​ACA​AGA-3′, Caspase 3 forward primer 5′-TGG​TTC​ATC​CAG​TCG​CTT​TG-3′ and reverse primer 5′-CAT​TCT​GTT​GCC​ACC​TTT​CG-3′, Caspase 9 forward primer 5′-CAG​GCC​CCA​TAT​GAT​CGA​GG-3′ and reverse primer 5′-GGC​CTG​TGT​CCT​CTA​AGC​AG-3′, β-actin forward primer 5′-GGG​AAA​TCG​TGC​GTG​ACA​TT-3′ and reverse primer 5′-GGA​ACC​GCT​CAT​TGC​CAT-3′. In addition, the fold change in expression was calculated using 2^−ΔΔCT^ method.

### Western blot analysis

SMMC-7721 cells were pretreated with LFE (0.5 mg/ml) for 36 h. Total protein was extracted and detected with BCA Protein Concentration Determination Kit (Meilun Biotechnology Co., Ltd.). Protein samples were separated by 8%–15% SDS-PAGE (80 V, for 30 min, and then 120 V, for 40 min) and transferred to the PVDF membrane in the transfer buffer at 120 V for 1–3 h. The membranes were blocked with 5% skim milk for 2 h at room temperature and also nurtured overnight at 4°C with primary antibodies against β-actin, PTEN, PI3K, p-PI3K, Akt, p-Akt, VEGFA and Caspase 9 (Proteintech, United States). After being rinsed three times with TBST, membranes were incubated 2 h at room temperature in secondary antibodies and subsequently be visualized with EasySee Western Blot Kit (Beijing Quanshi Gold Biotechnology Co., Ltd., Beijing, China). Finally, the images were quantified using ImageJ software and standardized against β-actin. In addition, three independent assays were performed.

### Statistical analysis

SPSS software (version 19.0) was employed to carry out most of the statistical analyses except metabonomics. The data were shown as mean ± SD. Statistical comparisons were explored using one-way analysis of variance (ANOVA) followed by the least significant difference (LSD) test. In addition, the difference was of statistical significance when values of *p* < 0.05, while it indicated very significant when *p* < 0.01.

## Results

### Chemical components absorbed into rat plasma

**FIGURE 1:**
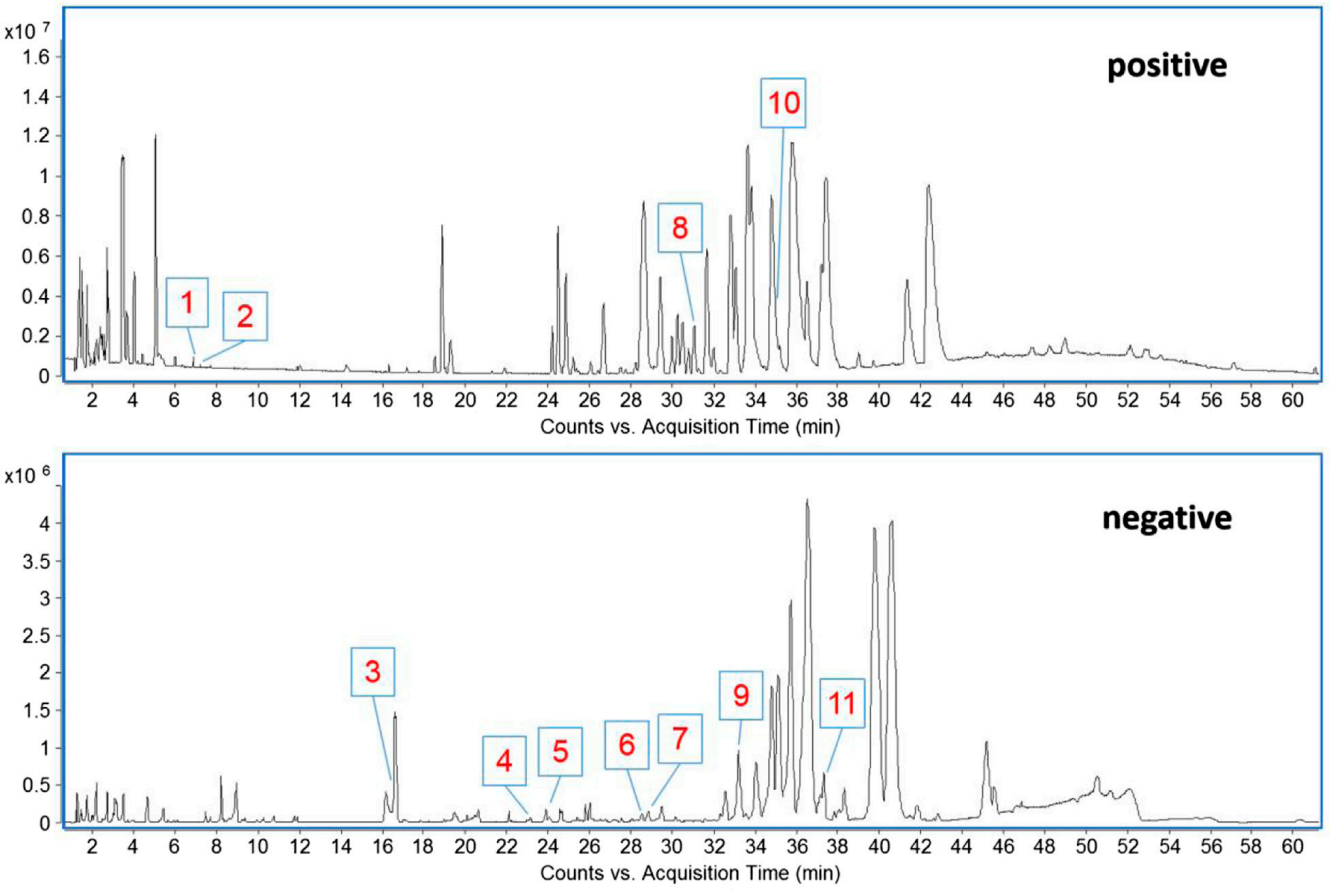
The base peak chromatogram (BPC) of blood components from LFE in positive and negative modes.

In the early stage, our research group separated and identified the chemical components contained in LFE, and identified totally 30 chemical components (Zhang et al., 2020). In the current work, the chemical components absorbed into rat plasma was further explored, and 11 prototype blood components were found, including phellopterin, 2-hydroxy-3-oxo-1,4(5)-oleanadien-28-oic acid, lantanoic acid and so on. In addition, eight triterpenoids, two coumarins and one phenolic components were included (Table 1) (Figure 1).

**TABLE 1:** Prototype blood component of LFE.

No.	Retention time (RT) (min)	Formula	Theoretical mass (m/z)	Measured mass (m/z)	Mass error (ppm)	Identified compounds
1	6.575	C_16_H_14_O_4_	293.0784 [M+Na]^+^	293.0755[M+Na]^+^	9.89[M+Na]^+^	Imperatorin
2	7.104	C_17_H_16_O_5_	301.1071[M+H]^+^	301.1063[M+H]^+^	2.65[M+H]^+^	Phellopterin
3	16.570	C_29_H_40_O_4_	473.3272[M-H]^−^	473.3303[M-H]^−^	−6.54[M-H]^−^	2-Hydroxy-3-oxo-1,4(5)-oleanadien-28-oic acid
4	23.033	C_30_H_44_O_5_	483.3116[M-H]^−^	483.3123[M-H]^−^	−1.44[M-H]^−^	Lantanoic acid
5	24.025	C_30_H_48_O_5_	487.3429[M-H]^−^	487.3433[M-H]^−^	−0.82[M-H]^−^	Arjunolic acid
6	28.752	C_30_H_46_O_4_	469.3323[M-H]^−^	469.3331[M-H]^−^	−1.70[M-H]^−^	6β-Hydroxyl-3-oxolup-20(29)-en-28-oic acid
7	29.000	C_30_H_44_O_4_	467.3167[M-H]^−^	467.3165[M-H]^−^	0.42[M-H]^−^	Liquidambaric lactone
8	31.650	C_29_H_44_O_4_	457.3312[M+H]^+^	457.3316[M+H]^+^	0.87[M+H]^+^	2α,3β-Dihydroxy-23-demethyloleanol-4(24)-12 (13)-diene-28-carboxylic acid
9	33.661	C_30_H_44_O_4_	467.3167[M-H]^−^	467.3167[M-H]^−^	0.00[M-H]^−^	3,6-Dion-20(29)-lupen-28-oic acid
10	34.975	C_15_H_22_O_2_	235.1693[M+H]^+^	235.1688[M+H]^+^	2.12[M+H]^+^	Sesquichamaenol
11	37.331	C_30_H_46_O_3_	453.3374[M-H]^−^	453.3387[M-H]^−^	−2.86[M-H]^−^	3-Oxo-ursolic acid

### Effect of liquidambaris fructus extract on physiological indexes of hepatocellular carcinoma rats

**FIGURE 2:**
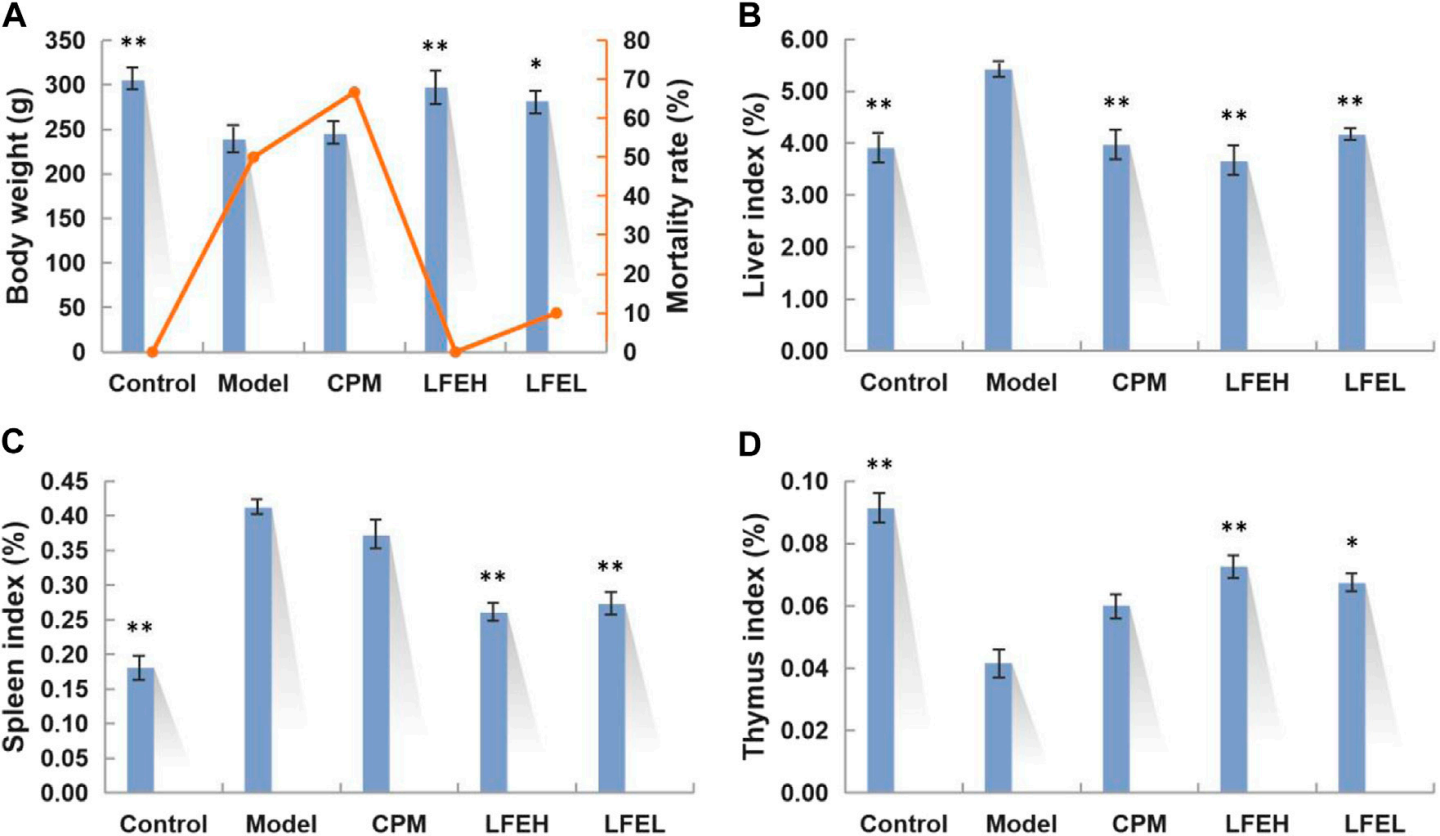
Effects of LFE on physiological indexes of HCC rats. **(A)** Body weight and mortality rate of different groups. **(B)** Liver index of different groups. **(C)** Spleen index of different groups. **(D)** Thymus index of different groups. The findings were denoted to be mean ± SE. *n* = 8. *p < 0.05, **p < 0.01 vs. model group. Control, the blank group; Model, the model group; CPM, the cyclophosphamide group; LFEH, LFE high-dose group; LFEL, LFE low-dose group.

In 1–6 weeks of modeling, rats in each group exhibited good appetites, which were active and lively with their hair being glossy and dense. After 6–16 weeks of modeling, rats in the model group suffered from anorexia, decreased diet, decreased activity, disordered and dull hair, and the mortality reached up to 50%. Rats in cyclophosphamide group presented less diet, severe depilation, tooth loss, weight loss and other symptoms with the mortality being higher (66.67%). In comparison with the model group, rats in LFE group were in relatively good physical condition and their hair was smooth and tidy with no severe depilation, tooth loss, weight loss and other symptoms. Compared with the model group and cyclophosphamide group, there was no death in LFE high-dose group, and the mortality in low-dose group was lower (10%). Additionally, at week 16, the body weight of rats in the blank group, LFE high-dose and low-dose groups was obviously higher than that in the model group (*p* < 0.01 or *p* < 0.05), while there existed no obvious difference between cyclophosphamide group and model group (Figure 2A). It can be seen that there exists a certain negative correlation between body weight and mortality. And also indicated that LFE could improve the quality of life and prolong the survival time of HCC rats.

The changes of some physiological indexes such as liver index, thymus index and spleen index can be used to assess the efficacy of drugs. The findings of this study demonstrated that the liver tissue of the model group was congested and inflamed, and its liver index was higher than that of the blank group (*p* < 0.01). Besides, the liver index of LFE high and low dose groups were lower than that of model group (*p* < 0.01), close to that of the cyclophosphamide and blank groups. When the body is cancerous, the immune function is lost, the thymus index decreases, and the spleen index increases. According to the results, compared with the blank group, the thymus index of the model group was smaller and the spleen index was larger, suggesting that the immune function of liver cancer rats was decreased. While there was no difference in thymus index and spleen index between cyclophosphamide group and model group (*p* > 0.05), indicating that although cyclophosphamide improved liver injury, it also reduced the immunity of the body, thus affecting the quality of life of rats. In comparison with the model group, the spleen indexes of LFE groups were smaller (*p* < 0.01) and the thymus indexes were larger (*p* < 0.01 or *p* < 0.05), suggesting that LFE could significantly increase the immune capacity of the body and therefore resist liver cancer, especially at high doses (Figures 2B–D).

### Effect of liquidambaris fructus extract on pathological indexes of liver tissue in rats with hepatocellular carcinoma

**FIGURE 3:**
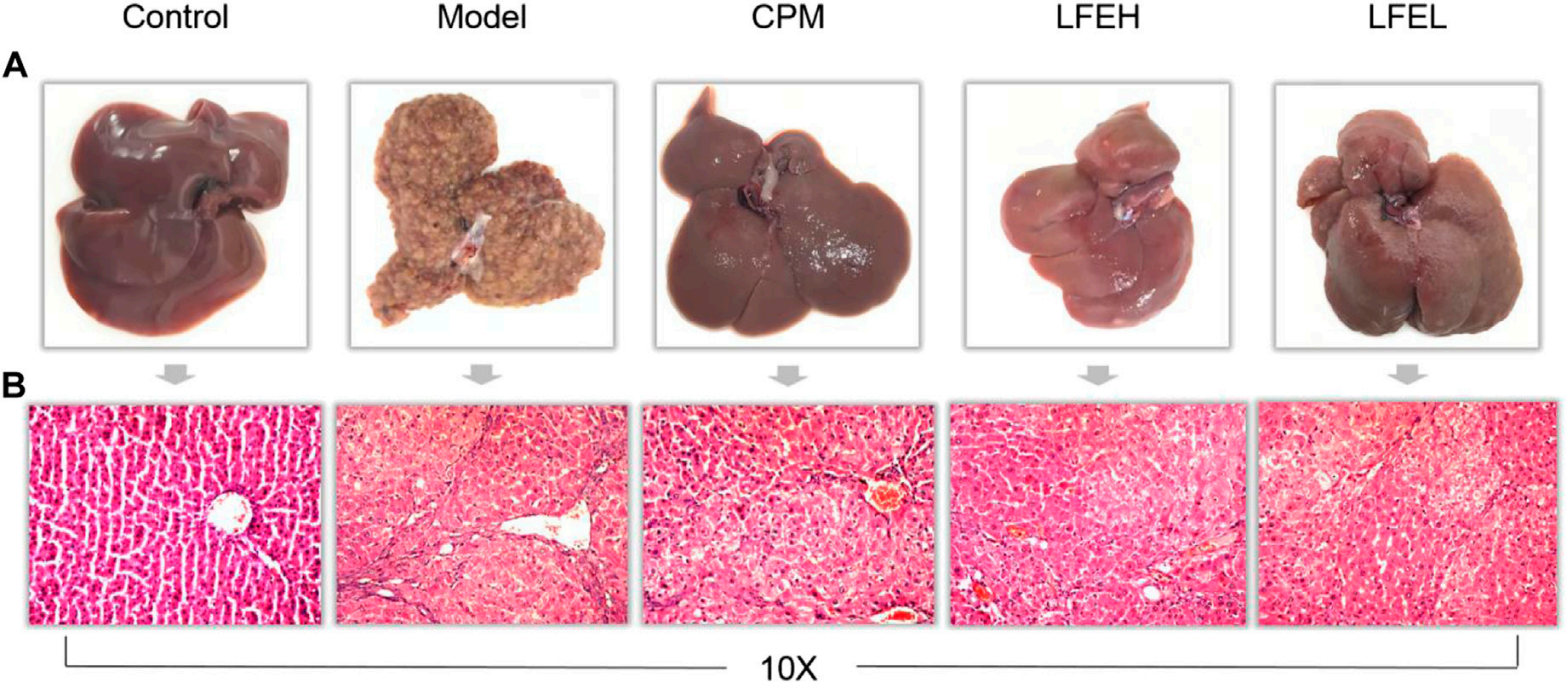
Impact of LFE on pathological indexes of liver tissue in rats with HCC. **(A)** Liver appearance of rats in each group. **(B)** Liver histopathology of rats in each group. Control, the blank group; Model, the model group; CPM, the cyclophosphamide group; LFEH, LFE high-dose group; LFEL, LFE low-dose group.

Based on the appearance, the liver tissue of the blank group was ruddy in color, smooth in surface and soft in texture. The liver tissue of rats in the model group was yellowish in color, and the surface was densely covered with white vesicular tumor including nodules. The nodules were large in volume, and the diameter of some tumors was over 1 mm. The texture was hard. In the low dose group of LFE, the surface of liver tissue was slightly rough and hard, accompanied by a small number of gray white nodules. However, in the high dose group, the surface of liver tissue was smooth, and some small pimple like protrusions appeared on the surface of liver, which was close to the blank group. In addition, the liver tissue of cyclophosphamide group was similar to that of normal group, with smooth surface and slightly dark color (Figure 3A).

According to the pathological section, the liver lobule structure of the blank group rats is complete and clear. In addition, hepatic cord is radially arranged from the central vein, the hepatocyte interface is clear, the size is basically the same, the arrangement is neat, the nucleus is large and round, the cytoplasm is uniform, the nucleolus is clear, the liver lobule structure is very complete, and there exists no significant inflammatory cell invasion. In the model group, the liver cells showed obvious atypical proliferation, increased heterotypic cells, irregular arrangement, deep staining due to nuclear division, hemorrhage, necrosis and inflammatory cell infiltration in some parts. When compared with model group, the hepatocytes of LFE and cyclophosphamide groups were arranged orderly, some hepatocytes were still arranged in a cord shape, the nodules were small and few, and the hepatocytes had less heterogeneous proliferation, which was found to be close to the liver tissue of the blank group. The high dose group of LFE was better than the low dose group, which could alleviate the degree of liver cell damage and have the trend of recovery to normal cells. The results proved that LFE possesses prominent anti-liver tumor effect (Figure 3B).

### Effect of liquidambaris fructus extract on biochemical indexes of serum in rats with hepatocellular carcinoma

**FIGURE 4:**
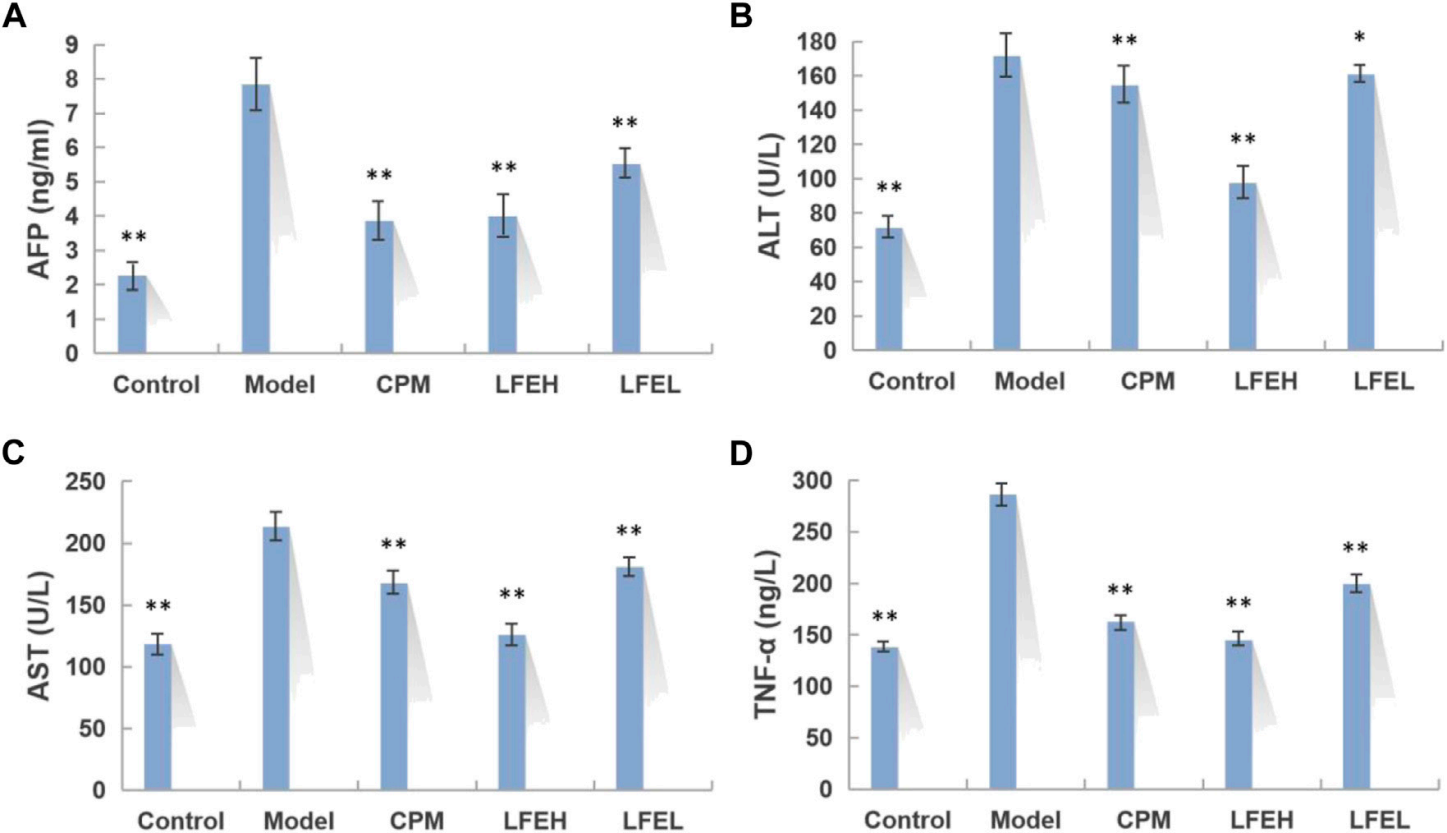
Impact of LFE on biochemical indexes of serum in rats with HCC. **(A)** Content of AFP in each group. **(B)** Content of ALT in each group. **(C)** Content of AST in each group. **(D)** Content of TNF-α in each group. The results were expressed as mean ± SE. *n* = 8. *p < 0.05, **p < 0.01 vs. model group. Control, the blank group; Model, the model group; CPM, the cyclophosphamide group; LFEH, LFE high-dose group; LFEL, LFE low-dose group.

AFP is a tumor biomarker of liver cancer, serum ALT and AST levels are characteristic indexes reflecting liver function, and tumor necrosis factor TNF-α is an important inflammatory factor. The changes of these indicators can also reflect the severity of HCC and the therapeutic effect of drugs from a biochemical point of view. The levels of AFP, ALT, AST, and TNF-α in serum of DEN-induced HCC rats after LFE intervention were determined by ELISA. In comparison with blank group, the contents of AFP, ALT, AST, and TNF-α in serum of model group were obviously enhanced (*p* < 0.01). However, in comparison with model group, the contents of AFP, ALT, AST, and TNF-α in serum of LFE high-dose group reduced obviously (*p* < 0.01). The levels of AFP, ALT, AST, and TNF-α in LFE low-dose group were also decreased in varying degrees (*p* < 0.01 or *p* < 0.05) (Figures 4A-D). The effect of high dose of LFE on serum AFP content of rats was similar to that of cyclophosphamide, the influences on ALT and AST were better than those of cyclophosphamide, and the impact on TNF-α was also similar to that of cyclophosphamide, slightly better, which indicated that LFE possesses preferable anti-tumor, hepatoprotective and anti-inflammatory effects.

### Analysis of metabolic profiles

**FIGURE 5:**
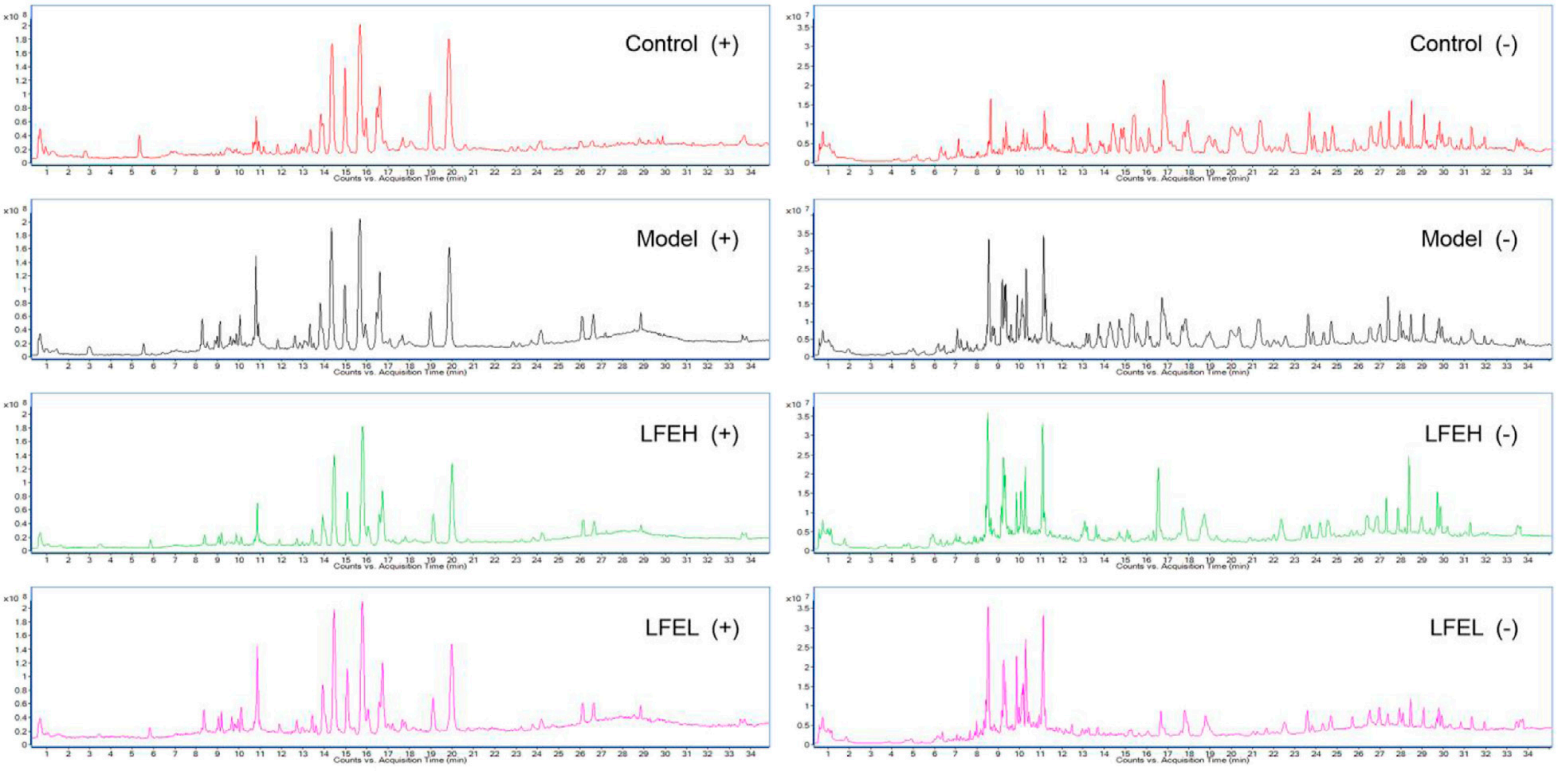
Metabolic profile of each group in positive and negative modes. Control, the blank group; Model, the model group; LFEH, LFE high-dose group; LFEL, LFE low-dose group.

**FIGURE 6:**
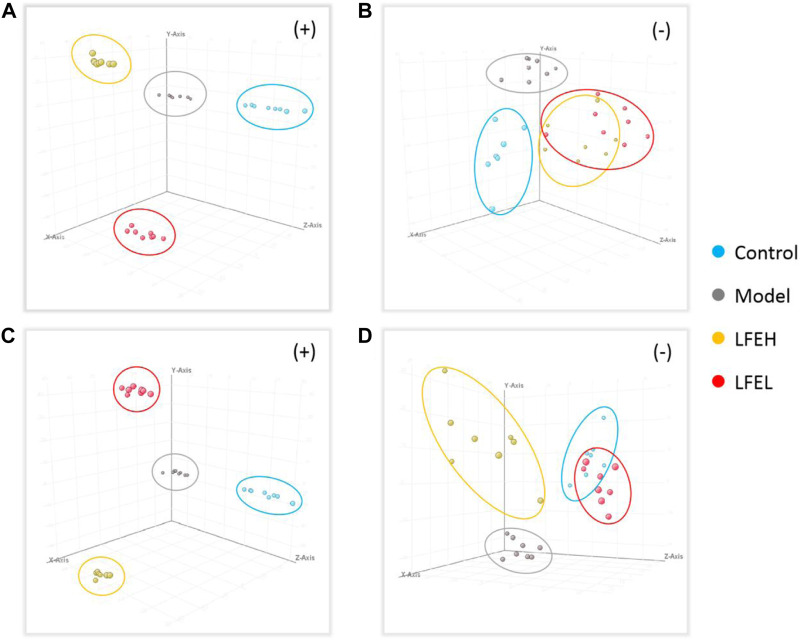
PCA and PLS-DA analysis. **(A)** PCA analysis in positive mode. **(B)** PCA analysis in negative mode. **(C)** PLS-DA analysis in positive mode. **(D)** PLS-DA analysis in negative mode. Control, the blank group; Model, the model group; LFEH, LFE high-dose group; LFEL, LFE low-dose group.

The metabolic profile of each group was obtained by UPLC-QTOF-MS analysis. The total ion chromatography (TIC) was shown in Figure 5. PCA and PLS-DA analysis results revealed that the points in the group are relatively clustered and have good homogeneity. Better separation can be achieved in the spatial position between each group, and both positive and negative ion modes are far away from the model group, indicating that the metabolites in the body are different (Figures 6A–D).

### Identification of endogenous differential metabolites

Based on the significant statistical analysis and the contribution degree of compounds, combined with clinical and biological significance, 82 compounds (59 from positive ion mode, 30 from negative, with 7 both from positive and negative) were preliminarily selected as endogenous differential metabolites. They were identified through the comparison of HMDB, Metlin, KEGG and other databases, as displayed in Tables 2, 3.

**TABLE 2:** Endogenous differential metabolites in positive ion mode.

No.	Retention time (RT) (min)	Endogenous differential metabolites	Formula	Measured mass (m/z)	Ion Mode	Trend	
						Control vs. Model	LFEH, LFEL vs. Model
1	0.673	Choline	C_5_H_14_NO	104.1067	[M+H]^+^	Down	Down
2	0.689	Carnitine	C_7_H_15_NO_3_	162.1124	[M+H]^+^	Up	Up
3	0.716	Valine	C_5_H_11_NO_2_	118.0859	[M+H]^+^	Up	Up
4	0.911	Asparagine	C_4_H_8_N_2_O_3_	155.0430	[M+Na]^+^	Up	Up
5	0.970	Acetylcarnitine	C_9_H_17_NO_4_	204.1228	[M+H]^+^	Up	Up
6	1.036	2-Phenylacetamide	C_8_H_9_NO	136.0758	[M+H]^+^	Down	Down
7	1.390	Leucine	C_6_H_13_NO_2_	132.1017	[M+H]^+^	Up	Up
8	1.433	Tyrosine	C_9_H_11_NO_3_	182.0805	[M+H]^+^	Down	Down
9	1.483	Isoleucine	C_6_H_13_NO_2_	132.1017	[M+H]^+^	Up	Up
10	2.971	L-Phenylalanine	C_9_H_11_NO_2_	166.0861	[M+H]^+^	Down	Down
11	4.745	Isobutyrylcarnitine	C_11_H_22_NO_4_	232.1528	[M+H]^+^	Up	Up
12	5.379	Indolelactic acid	C_11_H_9_NO_2_	188.0714	[M+H]^+^	Down	Down
13	5.379	Tryptophan	C_11_H_12_N_2_O_2_	205.0978	[M+H]^+^	Down	Down
				227.0795	[M+Na]^+^		
14	6.421	Valerylcarnitine	C_12_H_23_NO_4_	246.1704	[M+H]^+^	Up	Up
15	6.498	Pivaloylcarnitine	C_12_H_23_NO_4_	246.1694	[M+H]^+^	Up	Up
16	7.366	Hexanoylcarnitine	C_13_H_25_NO_4_	260.1849	[M+H]^+^	Up	Up
17	8.809	Octanoylcarnitine	C_15_H_29_NO_4_	288.2156	[M+H]^+^	Up	Up
18	8.311	Sulfoglycolithocholate	C_26_H_43_NO_7_S	514.2820	[M+H]^+^	Down	Down
				536.2643	[M+Na]^+^		
				478.2607	[M+H-2H_2_O]^+^		
				496.2712	[M+H-H_2_O]^+^		
19	8.889	Taurallocholic acid	C_26_H_45_NO_7_S	480.2760	[M+H-2H_2_O]^+^	Down	Down
				498.2864	[M+H-H_2_O]^+^		
20	8.972	Tauroursocholic acid	C_26_H_45_NO_7_S	480.2762	[M+H-2H_2_O]^+^	Down	Down
				498.2868	[M+H-H_2_O]^+^		
				516.2969	[M+H]^+^		
				538.2791	[M+Na]^+^		
				560.2628	[M+2Na-H]^+^		
21	9.584	Tetracosahexaenoic acid	C_24_H_36_O_2_	357.2776	[M+H]^+^	Down	Down
22	9.782	Glycocholic acid	C_26_H_43_NO_6_	430.2919	[M+H-2H_2_O]^+^	Down	Down
				448.3040	[M+H-H_2_O]^+^		
				466.3146	[M+H]^+^		
				488.2973	[M+Na]^+^		
23	9.881	12-Ketodeoxycholic acid	C_24_H_36_O_2_	373.2727	[M+H-H_2_O]^+^	Down	Down
				391.2830	[M+H-H_2_O]^+^		
24	10.030	7-Ketodeoxycholic acid	C_24_H_38_O_5_	371.2592	[M+H-2H_2_O]^+^	Down	Down
				389.2679	[M+H-H_2_O]^+^		
				407.2775	[M+H]^+^		
				428.2601	[M+Na]^+^		
25	10.658	Palmitic acid	C_16_H_32_O_2_	274.2737	[M+NH_4_]^+^	Down	Down
26	10.790	Nutriacholic acid	C_24_H_38_O_4_	355.2624	[M+H-2H_2_O]^+^	Down	Down
				373.2730	[M+H-H_2_O]^+^		
				391.2827	[M+H]^+^		
27	10.790	PI(16:0/18:0)	C_43_H_83_O_13_P	839.5623	[M+H]^+^	Down	Down
28	10.906	6,9,12,15,18,21-Tetracosahexaenoic acid	C_24_H_36_O_2_	357.2778	[M+H]^+^	Down	Down
29	11.154	Glycoursodeoxycholic acid	C_25_H_43_NO_5_	472.3011	[M+Na]^+^	Down	Down
				450.3188	[M+H]^+^		
30	11.501	Dodecanoylcarnitine	C_19_H_37_NO_4_	344.2779	[M+H]^+^	Up	Up
31	11.881	9,10-DHOME	C_18_H_34_O_4_	315.2521	[M+H]^+^	Down	Down
32	11.948	Leukotriene B	C_20_H_32_O_4_	337.2346	[M+H]^+^	Down	Down
33	12.295	Sphinganine	C_18_H_39_NO_2_	302.3049	[M+H]^+^	Down	Down
				324.2860	[M+Na]^+^		
34	12.460	Sphinganine 1-phosphate	C_18_H_38_NO_5_P	402.2364	[M+Na]^+^	Down	Down
				380.2547	[M+H]^+^		
35	12.625	LysoPC(14:0/0:0)	C_22_H_46_NO_7_P	490.2892	[M+Na]^+^	Up	Up
				468.3073	[M+H]^+^		
36	13.320	LysoPC(16:1(9z)/0:0)	C_24_H_48_NO_7_P	516.3046	[M+Na]^+^	Up	Up
				494.3228	[M+H]^+^		
37	13.683	Tetradecanoylcarnitine	C_21_H_41_NO_4_	394.2913	[M+Na]^+^	Up	Up
				372.3098	[M+H]^+^		
38	13.816	LysoPC(18:2(9z,12z))	C_26_H_50_NO_7_P	542.3206	[M+Na]^+^	Up	Up
				520.3392	[M+H]^+^		
39	13.915	LysoPC(20:4(5z,8z,11Z,14Z))	C_28_H_50_NO_7_P	566.3202	[M+Na]^+^	Up	Up
				544.3384	[M+H]^+^		
40	14.345	LysoPC(22:6(4z,7z,10Z,13Z,16Z,19Z))	C_30_H_50_NO_7_P	566.3202	[M+Na]^+^	Up	Up
				544.3384	[M+H]^+^		
41	14.510	Stearidonic acid	C_18_H_28_O_2_	277.2154	[M+H]^+^	Up	Up
42	14.956	LysoPC(16:0)	C_24_H_50_NO_7_P	518.3205	[M+Na]^+^	Up	Up
				496.3398	[M+H]^+^		
43	15.188	Eicosapentaenoic acid	C_20_H_30_O_2_	303.2307	[M+H]^+^	Up	Up
44	15.667	LysoPC(0:0/16:0)	C_24_H_50_NO_7_P	518.3203	[M+Na]^+^	Up	Up
				496.3403	[M+H]^+^		
45	15.932	LysoPC(18:1(9z))	C_26_H_52_NO_7_P	544.3361	[M+Na]^+^	Up	Up
				522.3540	[M+H]^+^		
46	16.444	LysoPC(18:1(11z))	C_26_H_52_NO_7_P	544.3361	[M+Na]^+^	Up	Up
				522.3545	[M+H]^+^		
				1,043.7036	[2M+H]^+^		
47	16.527	PC(14:0/16:0)	C_38_H_76_NO_8_P	688.5187	[M+H-H_2_O]^+^	Up	Up
48	17.139	L-Palmitoylcarnitine	C_23_H_45_NO_4_	400.3404	[M+H]^+^	Up	Up
49	17.684	LysoPC(20:2(11Z,14Z))	C_28_H_54_NO_7_P	570.3513	[M+Na]^+^	Up	Up
				548.3692	[M+H]^+^		
50	17.684	LysoPE(0:0/20:0)	C_25_H_52_NO_7_P	532.3374	[M+Na]^+^	Up	Up
				509.3481	[M+H]^+^		
51	18.036	2-Hydroxyhexadecanoylcarnitine	C_25_H_47_NO_4_	426.3502	[M+H]^+^	Up	Up
52	18.660	Docosapentaenoic acid	C_22_H_34_O_2_	331.2629	[M+H]^+^	Up	Up
53	18.990	LysoPC(18:0)	C_26_H_54_NO_7_P	546.3513	[M+Na]^+^	Up	Up
				524.3701	[M+H]^+^		
54	19.866	LysoPC(0:0/18:0)	C_26_H_54_NO_7_P	546.3516	[M+Na]^+^	Up	Up
				524.3714	[M+H]^+^		
55	20.577	LysoPC(20:1(11Z))	C_28_H_56_NO_7_P	550.3853	[M+H]^+^	Up	Up
				572.3669	[M+Na]^+^		
56	20.941	Stearoylcarnitine	C_25_H_49_NO_4_	428.3712	[M+H]^+^	Up	Up
57	23.156	Docosahexaenoic acid	C_22_H_32_O_2_	329.2464	[M+H]^+^	Up	Up
58	23.735	Arachidonic acid	C_20_H_32_O_2_	305.2464	[M+H]^+^	Down	Down
59	26.573	Cer(d18:0/12:0)	C_30_H_61_NO_3_	484.4714	[M+H]^+^	Up	Up

**TABLE 3:** Endogenous differential metabolites in negative ion mode.

No.	Retention time (RT) (min)	Endogenous differential metabolites	Formula	Measured mass (m/z)	Ion mode	Trend	
						Control vs. Model	LFEH, LFEL vs. Model
1	0.564	L-Lysine	C_6_H_14_N_2_O_2_	145.0965	[M-H]^−^	Up	Up
2	1.176	N-Acryloylglycine	C_5_H_7_NO_3_	128.0339	[M-H]^−^	Down	Down
3	4.003	L-Phenylalanine	C_9_H_11_NO_2_	164.0700	[M-H]^−^	Down	Down
4	6.846	(R)-3-Hydroxyhexanoic acid	C_6_H_12_O_3_	131.0695	[M-H]^−^	Up	Up
5	7.061	p-Cresol sulfate	C_7_H_8_O_4_S	187.0054	[M-H]^−^	Up	Up
6	8.549	Taurallocholic acid	C_26_H_45_NO_7_S	512.2692	[M-H]^−^	Down	Down
7	9.277	Tauroursocholic acid	C_26_H_45_NO_7_S	514.2809	[M-H]^−^	Down	Down
8	9.491	Tauroursodeoxycholic acid	C_26_H_45_NO_6_S	498.2860	[M-H]^−^	Down	Down
9	11.128	Cholic acid	C_24_H_40_O_5_	815.5652	[2M-H]^−^	Down	Down
				407.2778	[M-H]^−^		
10	11.227	Isohyodeoxycholic acid	C_24_H_40_O_4_	391.2823	[M-H]^−^	Up	Up
				783.5737	[2M-H]^−^		
11	11.492	Nutriacholic acid	C_24_H_38_O_4_	389.2666	[M-H]^−^	Down	Down
				779.5423	[2M-H]^−^		
12	13.162	Sphingosine 1-phosphate	C_18_H_38_NO_5_P	378.2382	[M-H]^−^	Down	Down
				757.4850	[2M-H]^−^		
13	13.294	Deoxycholic acid	C_24_H_40_O_4_	391.2817	[M-H]^−^	Down	Down
				783.5731	[2M-H]^−^		
14	13.707	Chenodeoxycholic acid	C_24_H_40_O_4_	391.2819	M+FA-H	Down	Down
				437.2870	[M-H]^−^		
				783.5731	[2M-H]^−^		
15	14.716	LysoPE(20:2(11Z,14Z)/0:0)	C_25_H_48_NO_7_P	504.3055	[M-H]^−^	Up	Up
16	14.815	LysoPE(0:0/22:4(7Z,10Z,13Z,16Z))	C_27_H_48_NO_7_P	528.3048	[M-H]^−^	Up	Up
17	15.278	LysoPE(0:0/20:2(11Z,14Z))	C_25_H_48_NO_7_P	504.3057	[M-H]^−^	Up	Up
18	15.360	LysoPE(22:4(7Z,10Z,13Z,16Z)/0:0)	C_27_H_48_NO_7_P	528.3052	[M-H]^−^	Up	Up
19	16.022	LysoPE(18:0/0:0)	C_23_H_48_NO_7_P	480.3055	[M-H]^−^	Up	Up
20	16.732	LysoPC(15:0)	C_23_H_48_NO_7_P	480.3055	[M-H]^−^	Up	Up
21	16.848	LysoPE(0:0/18:0)	C_23_H_48_NO_7_P	480.3055	[M-H]^−^	Up	Up
22	17.658	LysoPE(20:1(11Z)/0:0)	C_25_H_50_NO_7_P	506.3209	[M-H]^−^	Up	Up
23	17.791	LysoPE(0:0/20:1(11Z))	C_25_H_50_NO_7_P	506.3209	[M-H]^−^	Up	Up
24	20.006	LysoPI(18:0/0:0)	C_27_H_53_O_12_P	599.3158	[M-H]^−^	Up	Up
25	21.262	LysoPE(20:0/0:0)	C_25_H_52_NO_7_P	508.3367	[M-H]^−^	Up	Up
26	21.957	LysoPE(22:1(13Z)/0:0)	C_27_H_54_NO_7_P	534.3514	[M-H]^−^	Up	Up
27	22.188	LysoPC(17:0)	C_25_H_52_NO_7_P	508.3367	[M-H]^−^	Up	Up
28	22.552	Glycocholic acid	C_26_H_43_NO_6_	464.3104	[M-H]^−^	Up	Up
29	23.825	Docosahexaenoic acid	C_22_H_32_O_2_	327.2299	[M-H]^−^	Down	Down
30	24.337	Arachidonic acid	C_20_H_32_O_2_	303.2301	[M-H]^−^	Down	Down

### Effect on the expression of PI3K-Akt signal pathway related genes

**FIGURE 7:**
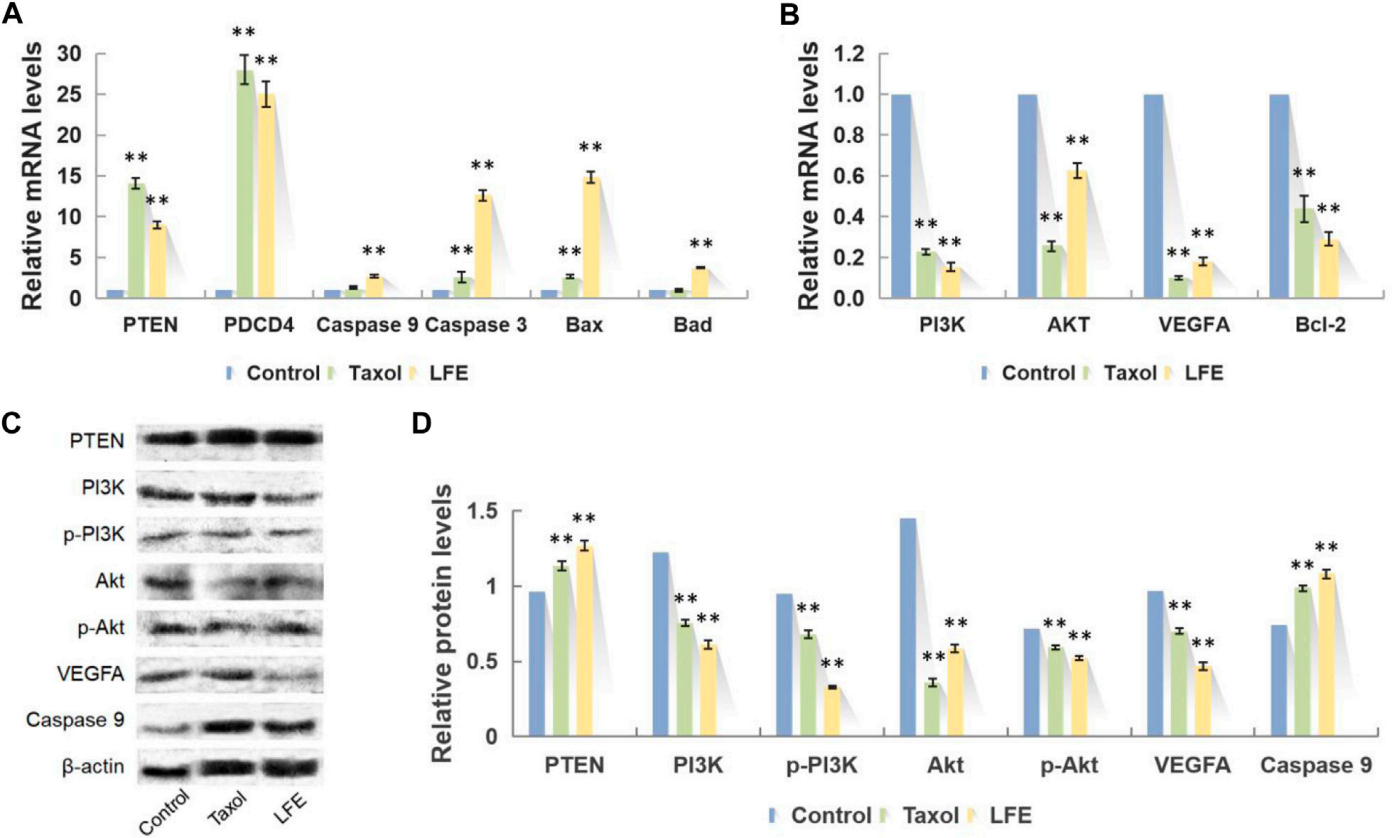
Representative photographs of q-PCR and Western blot. **(A)** Upregulated mRNA after LFE treatment. **(B)** Downregulated mRNA after LFE treatment. **(C)** Representative western blots showing PTEN, PI3K, p-PI3K, Akt, p-Akt, VEGFA and Caspase 9 after LFE treatment. **(D)** Relative protein levels after LFE treatment.

In comparison with the blank group, LFE regulated the mRNA expression levels of PTEN, PI3K, AKT, PDCD4, VEGFA, Caspase 9, Caspase 3, Bcl-2, Bax and Bad in liver cancer cells to different degrees. Among them, the relative expressions of PTEN, PDCD4, Caspase 9, Caspase 3, Bax and Bad genes were upregulated, whereas those of PI3K, AKT, VEGFA and Bcl-2 genes were downregulated (Figures 7A,B).

### Effect on the expression of PI3K-Akt signal pathway related proteins

Compared with the blank group, LFE regulated the proteins expression levels of PTEN, PI3K, p-PI3K, Akt, p-Akt, VEGFA and Caspase 9 in liver cancer cells to different degrees. Among them, the relative expressions of PI3K, p-PI3K, Akt, p-Akt and VEGFA proteins presented downregulation (*p* < 0.01), while that of PTEN and Caspase 9 showed upregulation (*p* < 0.01) (Figures 7C,D).

## Discussion

Liver cancer refers to one of the top ten cancers globally in terms of incidence rate and mortality (Bao et al., 2017). In addition, it is also the most common malignant tumor in China, which seriously affects people’s lives. At present, liver cancer is not sensitive to radiotherapy and chemotherapy drugs, and clinical treatment urgently needs an alternative or auxiliary alternative drug (Guo et al., 2019). Previous studies of our research group have found that LFE exhibits perfect inhibitory impact on liver tumor cells and induces cell cycle arrest and apoptosis (Zhang et al., 2020). On this basis, this work carried out studies on the blood components, pharmacological effects and action mechanism of LFE *in vivo*, with the purpose of further revealing its pharmacodynamic material basis and mechanism of action.

In this study, through the research method of serum pharmacochemistry (Qian et al., 2021), the blood inflow components of rats’ plasma after intragastric administration of LFE were analyzed. Meanwhile, 11 prototype components, including imperatorin, phlopterin, arjunolic acid and liquidambaric lactone were found. According to relevant literature reports, imperatorin has the ability to cause Mcl-1 degradation, which then releases Bak and Bax and activates the intrinsic apoptosis pathway, causing multidrug-resistant liver cancer cells to undergo apoptosis (Li et al., 2014). It can reverse the drug resistance in cisplatin-resistant liver cancer cells with cisplatin treatment *in vitro* (Hu et al., 2015). Ajianolic acid possesses anti-inflammatory and anti-tumor cell proliferation activities, and exerts protective effects on liver and kidney toxicity induced by cisplatin (Nehal et al., 2016; Sherif, 2021). Some studies have shown that ajianolic acid possesses nano-sized self-assembly characteristics, is easy to be penetrated by cancer cells, and is non-toxic to normal cells (Manna et al., 2020). Liquidambaric lactone features anti-angiogenic properties, which can significantly inhibit VEGF-induced proliferation of HUVECs endothelial cells and effectively lower VEGF-induced cell migration (Zhu et al., 2021). 3-Oxo-ursolic acid is cytotoxic to a variety of tumor cells, exhibiting potent cytotoxic activities both in murine and in human cancer cell lines (Min et al., 2000). In addition, phellopterin (Sumiyoshi et al., 2014), 3,6-dion-20(29)-lupen-28-oic acid (Zhang et al., 2020) and others were reported to have varying degrees of antitumor activity. The above studies proved that the monomer components of LFE are certainly the active substances against HCC *in vivo*.

Based on the DEN-induced rat liver cancer model, this study investigated the anti-tumor efficacy of different doses of LFE. After a 4-month experimental cycle, it was found that the survival state of rats in LFE administration groups was significantly better than that in model group. Compared with the mortality of 50% in the model group, there was no death in LFE high-dose administration group, while the mortality of the positive drug cyclophosphamide was 66.67%. Thus, it is demonstrated that LFE has a definite effect on improving the survival cycle in the treatment of HCC. Furthermore, the body weight of rats in LFE high-dose group was also close to the blank group, which was obviously higher than that in model and cyclophosphamide groups. Liver index, spleen index, thymus index and other physiological indicators performed well. The above experimental results proved that LFE can inhibit the abnormal proliferation of liver cells, reduce inflammatory infiltration of liver cells and lower liver body ratio, so as to exert the role of repairing liver function. At the same time, LFE can also increase thymus index, decrease spleen index, and enhance the immune function of liver cancer rats. Thus, it is shown that LFE has a clear role in improving the quality of life in the treatment of HCC. From the perspective of pathological indicators, LFE high-dose group can significantly improve the degree of liver cell injury, with a trend of recovery to normal cells. The levels of liver cancer biomarker AFP, liver injury indicators ALT, AST and pro-inflammatory factor TNF-α in serum of rats treated with LFE were significantly lower than those in model group and close to blank group. It is demonstrated that LFE has definite anti-tumor, hepatoprotective and anti-inflammatory impact on the treatment of HCC. Pharmacological and pharmacodynamic experiments based on DEN-induced rat liver cancer model proved that LFE could prolong the life cycle and improve the quality of life, thus treating liver tumor.

The occurrence and development of HCC refers to a complex multi-step process involving multiple genes and multiple pathways. As one of the most frequently activated signaling pathways in human cancer, the PI3K/Akt signaling pathway mediates almost 50% of malignant tumors. PI3K/Akt signaling pathway plays a pivotal role in intracellular and extracellular signal transduction. As an important regulatory pathway in the development and progression of HCC, PI3K/Akt signaling pathway is closely associated with the proliferation, apoptosis, autophagy, invasion and migration of HCC cells (Martini et al., 2014). In the previous experimental study, the research group found that triterpenoids in LF can regulate the PI3K/Akt signaling pathway to promote the apoptosis of HCC cells (Zhang et al., 2020). In order to further verify the rationality of the discovered mechanism and explore the in-depth mechanism of LFE in anti-tumor, hepatoprotective, anti-inflammatory and other pharmacological effects, this study adopted non-target metabolomics combined with q-PCR and Western blot technology for investigating the key targets and pathways of LFE.

Through the mechanism study of non-target metabolomics, it was found that endogenous lipid metabolites including sphingosine-1-phosphate (S1P), sphingonine 1-phosphate and sphingonine changed, in which S1P has emerged as an important signaling molecule that has been discovered to be involved in many cellular functions (Nagahashi et al., 2015), containing promoting cell proliferation, death, aging, adhesion, migration, angiogenesis and inflammation. These functions are mediated by G protein-coupled S1P receptors, where S1P(1) stimulates cell proliferation through a G(i) mediated signaling pathway including PI3K/Akt and ERK, while S1P(2) mediates cell proliferation through G(12/13)/Rho/Rho kinase/PTEN-dependent Akt inhibition mechanism (Takuwa et al., 2012). In this study, LFE can downregulate the content of S1P in serum of HCC rats, thereby influencing the PTEN/PI3K/Akt signaling pathway. PTEN can inhibit the phosphorylation of the intracellular protein PI3K and downregulate the activity of phosphatidylinositol triphosphate (PIP3), leading to the inhibition of AKT recruitment and phosphorylation on the cell membrane, and affecting the protein proportion on the mitochondrial membrane (Carnero et al., 2008). Bax binds with Bcl-2 to form apoptotic dimer. In addition, it promotes the release of cytochrome C and apoptosis-inducing factors, promotes the occurrence of Caspase cascade reaction, and triggers apoptosis through endogenous pathway. Based on q-PCR and Western blot experiments, it was found that LFE could upregulate PTEN gene and protein expression, inhibit the phosphorylation of anti-apoptotic proteins PI3K and Akt, upregulate the level of Bad gene and downregulate the level of Bcl-2. Meanwhile, it promotes the activation of Caspase 9, the direct target protein downstream of Akt, further activates the downstream Caspase 3 protein, and finally inhibits the proliferation of liver cancer cells and causes apoptosis of liver cancer cells. In addition, the combination of VEGF and VEGFR1 will also affect Akt signaling pathway, generating tumor cell migration and invasion (Peng et al., 2021). This study found that LFE can downregulate the expression of VEGFA protein, thus inhibiting the angiogenesis of liver tumors and hindering the migration and invasion of tumor cells.

Besides, the differential endogenous substances eicosapentaenoic acid (EPA), docosahexaenoic acid (DHA), docosapentaenoic acid, 6,9,12,15,18,21-tetracosahaxaenoic acid and so on belong to polyunsaturated fatty acids (PUFA). EPA and DHA belong to n-3 polyunsaturated fatty acids, which are vulnerable to free radical attack and eventually lead to the formation of lipid peroxides. However, the main function of lipid peroxide is to inhibit DNA synthesis, hinder cell division and proliferation, and induce apoptosis (Huang et al., 2016). In this study, the contents of EPA and DHA in plasma of HCC rats treated with LFE were significantly higher than those of model group, providing that LFE can inhibit and block HCC cell cycle and induce apoptosis of HCC cells by increasing the content of unsaturated fatty acids.

Tumors are often accompanied by inflammatory response, and PI3K/Akt pathway also plays an important role in regulating inflammatory response. When PI3K/Akt pathway is activated, activated Akt can enhance the phosphorylation and degradation of NF-κB inhibitory protein IκB kinase, then lead to the activation of NF-κB, further induce the expression of TNF-α and other proinflammatory factors, and eventually lead to inflammatory response (Shi et al., 2016). In this study, the PI3K/Akt pathway was inhibited, the content of proinflammatory factor TNF-α in the blood of rats was decreased, and the content of endogenous differential metabolite arachidonic acid was also significantly lower than that of the model group, suggesting that LFE could also play an anti-tumor role by reducing inflammation.

Moreover, endogenous differential metabolites, such as LysoPC(14:0/0:0), LysoPC(16:1(9z)/0:0), and LysoPA(19:0/0:0), are related products of fatty acid metabolism. Liver is the main part of lipid metabolism and exerts an important role in maintaining the metabolic balance of blood lipids. The endogenous differential metabolite lysophosphatidylcholine (LPC) generates phosphatidylcholine (PC) under the action of phosphatidylcholine acyltransferase 1 (Lpcat1), PC loses the fatty acid at sn-2 position under the action of phospholipase A2 (PLA2), and generates LPC, which is converted into lysophosphatidic acid (LPA) under the catalysis of hemolytic phospholipase D. When liver cancer occurs in the body, lecithin cholesterol acyltransferase (LCAT) synthesis decreases and the level of Lyso PC in blood lowers significantly (Guri et al., 2017). In this study, compared with the model group, LFE can significantly increase the level of Lyso PCs in rat serum, also increasing LCAT synthesis to protect hepatocytes.

## Conclusion

To conclude, LFE possesses definite anti-liver cancer activity in physiology, pathology, biochemistry and other aspects. It can inhibit the growth of tumor cells, promote tumor cell apoptosis, reduce inflammatory reaction, protect hepatocytes, improve body immunity, improve the survival state of tumor rats, and prolong the life cycle. Besides, this study revealed the material basis of LFE, and further demonstrated that most of the above effects were related to the influence of PTEN/PI3K/Akt, fatty acid metabolism and other key signaling pathways from the perspective of metabolomics, which provide a scientific explanation for the clinical application of LFE. However, how the active ingredients are distributed, metabolized, and excreted in the body needs deeply explored, and the influence of LFE on other metabolic pathways will be further verified from the perspective of genes and proteins. These tasks will be the focus of our subsequent studies.

## Data Availability

The original contributions presented in the study are included in the article/supplementary material, further inquiries can be directed to the corresponding authors.

## Abbreviations

AFP, alpha-fetoprotein; ALT, alanine transaminase; AST, aspartate amino-transferase; BPC, base peak chromatogram; CPM, cyclophosphamide group; DHA, docosahexaenoic acid; EPA, eicosapentaenoic acid; HCC, hepatocellular carcinoma; LCAT, lecithin cholesterol acyltransferase; LF, liquidambaris fructus; LFE, liquidambaris fructus extract; LFEH, LFE high-dose group; LFEL, LFE low-dose group; LPA, lysophosphatidic acid; LPC, lysophosphatidylcholine; Lpcat1, phosphatidylcholine acyltransferase 1; LSD, least significant difference; MPP, Mass Profiler Professional; PC, phosphatidylcholine; PCA, principal component analysis; PIP3, phosphatidylinositol triphosphate; PLA2, phospholipase A2; PLS-DA, partial least squares discriminant analysis; PUFA, polyunsaturated fatty acids; S1P, sphingosine-1-phosphate; SD, Sprague-Dawley; TCM, traditional Chinese medicine; TIC, total ion chromatography; TNF-α, tumor necrosis factor-α; WHO, World Health Organization.
